# Baicalin–Zinc Complex Alleviates Inflammatory Responses and Hormone Profiles by Microbiome in Deoxynivalenol Induced Piglets

**DOI:** 10.3389/fnut.2021.738281

**Published:** 2021-10-08

**Authors:** Andong Zha, Ruiqi Tu, Zhijuan Cui, Ming Qi, Simeng Liao, Jing Wang, Bie Tan, Peng Liao

**Affiliations:** ^1^Chinese Academy of Sciences, Institute of Subtropical Agriculture, Changsha, China; ^2^College of Resource and Environment, University of Chinese Academy of Sciences, Beijing, China; ^3^College of Veterinary Medicine, Northwest A & F University, Yangling, China; ^4^Department of Animal Science and Technology, Hunan Agricultural University, Changsha, China

**Keywords:** baicalin-zinc complex, deoxynivalenol, intestinal microbiome, hormone secretion, inflammatory responses, weaned piglets

## Abstract

This study aimed to investigate the beneficial effect of baicalin–zinc complex (BZN) on intestinal microorganisms in deoxynivalenol (DON)-challenged piglets and the association between intestinal microorganisms and host immunity and hormone secretion. Forty weaned piglets were randomly divided into four treatments with 10 piglets in each treatment: (1) control (Con) group (pigs fed basal diet); (2) DON group (pigs fed 4 mg DON/kg basal diet); (3) BZN group (pigs fed 0.5% BZN basal diet); and (4) DBZN group (pigs fed 4 mg DON/kg and 0.5% BZN basal diet). The experiment lasted for 14 days. The BZN supplementation in DON-contaminated diets changed the intestinal microbiota composition and increased intestinal microbial richness and diversity of piglets. The BZN supplementation in DON-contaminated diets also alleviated the inflammatory responses of piglets and modulated the secretion of hormones related to the growth axis. Moreover, microbiota composition was associated with inflammatory and hormone secretion. In conclusion, BZN alleviated inflammatory response and hormone secretion in piglets, which is associated with the intestinal microbiome.

## Introduction

Deoxynivalenol (DON), originally known as vomitoxin, can cause vomiting, diarrhea, anorexia, neurological disorders, and immune dysfunction in humans and animals (1, 2). DON was reported to be the most common food-related mycotoxins all over the world (3–5). A study shows that DON was detected in 73 and 92% of wheat and corn in the USA (6). In another study in China, the detection rate of DON in corn was 93.2%, and the average concentration was 1,356.9 μg/kg (7). The DON detection rate in each region is shown as follows: East Asia (84.8%), Northern Europe (74.2%), Central America (70.0%), Central Europe (69.8%), North America (64.1%), South Africa (63.2%), Eastern Europe (59.9%), Southern Europe (52.9%), sub-Saharan Africa (49.5%), Middle East/North Africa Region (47.8%), Southeast Asia (42.5%), Oceania (34.5%), South America (26.9%), and South Asia (23.1%) (8). Pigs are very sensitive to the toxic effects of DON, and the long-term consumption of DON in pig can delay growth and reduce immune performance (9, 10). Therefore, repairing intestinal damage induced by DON is an important subject in the livestock production.

At low DON concentrations, DON promotes the production of immune factors, thereby increasing the risk of chronic immune disease or infection susceptibility (11, 12). DON induces secretion of serum immunoglobulin M (IgM), immunoglobulin A (IgA), immunoglobulin E (IgE), and immunoglobulin G (IgG) in mice or farm animals (13–15). DON induces a significant increase in tumor necrosis factor-α (TNF-α), interleukin-8 (IL-8), interleukin-1α (IL-1α), interleukin-1β (IL-1β), and gene expression in porcine intestinal epithelial cells (IPEC-1 cell line) (16). Furthermore, some studies also showed that DON can interfere with immune response by altering intestinal microbiome balance (17, 18). Previous studies reported that the growth retardation induced by DON is associated with the secretion of satiety hormones, such as peptide YY (PYY), cholecystokinin (CCK), and 5-hydroxytryptamine (5-HT), and growth hormone (GH) (19, 20). Therefore, we intend to develop a feed additive to reduce the toxic effects of DON. Baicalin–zinc complex (BZN) is a complex of baicalin and zinc. Although there are few studies on BZN at present, it has been proved to have good anti-inflammatory and antioxidant properties, which suggest that it may relieve the toxic effects of DON in pigs (21, 22). Moreover, zinc is an essential trace element for animals. It is involved in a multitude of body functions, ranging from the metabolism of nutrients to bone development, and as an activator to mediate the immune function of pigs (23, 24). It is generally added at 2,000 mg/kg in swine production to reduce diarrhea and promote pig growth. Baicalin (5,6-dihydroxy-2-phenyl-4H-1-benzopyran-4-one-7-O-D-β-glucuronic acid) is an extract from *Scutellaria baicalensis Georgi* and *Oroxylum indicum (L.) Kurz*, and it is commonly used in the cure of gastrointestinal infections and inflammatory diseases (25, 26). The dietary baicalin supplementation is tightly correlated to the intestinal microbiota composition, and it was also reported that baicalin has good antioxidant and anti-inflammatory in some studies (26–28).

This study aimed to investigate the beneficial effect of BZN on intestinal microbiome, inflammatory responses, and hormone profiles in DON-challenged piglets and the association between intestinal microbiome and host immunity and hormone secretion.

## Materials and Methods

### DON-Contaminated Diet and BZN Synthesis

Basal diet inoculate with *Fusarium graminearum* R6576 was fermented for 14 days to form DON-contaminated diet as described by Wu et al. (29). *F. graminearum* R6576 was supplied by Huazhong Agricultural University and preserved by Institute of Subtropical Sciences, Chinese Academy of Sciences (30). Baicalin was fully dissolved in 1% sodium bicarbonate solution, then equal molar ratio zinc sulfate was added to baicalin solution. After completion of the reaction, the precipitation was BZN. The dose of BZN and DON was referred to the previous literature (31).

### Animals, Diets, and Experimental Design

Forty weaned piglets with an average weight of 6.13 ± 0.42 kg (Landrace × Yorkshire, 21 days of age) were individually housed in a single column at the New wellful pig farm (New Wellful Co., Ltd, Hunan, China). Piglets were randomly divided into four diets, and 10 pigs were fed to each diet. The four groups were as follows: (1) control (Con) group (basal diet); (2) DON group (4 mg DON/kg basal diet); (3) BZN group (0.5% BZN basal diet); and (4) DBZN group (4 mg DON/kg and 0.5% BZN basal diet). The composition and nutrient level of the basal diet in this experiment is shown in Supplementary Table 1, and it meets nutrient requirements of pigs according to National Research Committee (NRC) (32, 33). All pigs were acclimated to the room for 3 days before the experiment, and experimental diets were provided in four equal daily meals at 7:30, 11:00, 14:30, and 18:00 for 14 days. Piglets housed on a 12-h light-dark cycle with free access to water, and the barn temperature was maintained at 30°C. Randomly selected seven pigs from each group for sampling after slaughter. After blood was collected by blood vessel, it was placed at room temperature for 1 h and centrifuged at 3,000 R for 15 min. The pale yellow liquid obtained was the serum. The intestine was opened in the middle ileum, and an appropriate amount of chyme was collected in a 50-ml sterile centrifugal tube. The collected serum and ileal chyme samples were quickly stored in liquid nitrogen. Serum and ileum chymes were stored at −80°C. The experiment was carried out under the supervision of the experimental animal ethics committee of the Institute of Subtropical Agriculture (Changsha, Human Province, China) (29, 34).

### 16s rRNA Sequencing

The 16s rRNA analysis of ileal chyme was conducted by Novogene Co., Ltd (Beijing, China) (35). The total DNA from ileal chyme was extracted by the CTAB/SDS method, and the DNA concentration and quality met the experimental requirements. Then, diluted the DNA to 1 mg/ml with sterile water. PCR was performed with specific primers as forward primer: ACTCCTACGGGAGGCAGCAG and reverse primer: GGACTACHVGGGTWTC TAAT (amplification of 16srRNA gene V3–V4 region); the PCR amplification products were detected and purified using a 2% agarose gel, then purified amplicons were used for the sequencing library. After the library has passed the quality assessment, it was sequenced on Ion S5TMXL (Thermo Fisher Scientific Co., Ltd., Waltham, CA, USA).

### Bioinformatics Analysis

Raw reads were demultiplexed and quality-filtered as previously described (36). Operational taxonomic units (OTUs) were clustered with 97% similarity for the effective tags of all samples by using the UPARSE software (version 7.0.1001), then used the Mothur method (https://www.mothur.org/) and SILVA database to annotate the species at the level phylum, order, family, genus, and class. The R software (version 2.15.3) was used to draw principal component analysis (PCA) chart, principal co-ordinates analysis (PCoA) chart and heatmap. Alpha diversity was used to analyze the diversity of intestinal microbiome. Linear discriminant analysis effect size (LEfSe) analysis was used galaxy module [linear discriminant analysis (LDA) score >2.5].

### Serum Indices (Immunoglobulins, Cytokines, Biochemical Indices, and Hormones Profiles)

Serum immunoglobulins: IgM, IgG, IgA, cytokines: TNF-α, IL-2, IFN-γ, IL-6, IL-1β, and hormones: 5-HT, GH, PYY, LEP, somatostatin (SST), insulin (INS), neuropeptide Y (NPY), insulin-like growth factor-1 (IGF-1), proopiomelanocortin (PMOC), agouti-related protein (AGRP), and glucagon-like peptide 1 (GLP-1) were determined using the commercially available ELISA kits from Nanjing Jiancheng Co., Ltd. (Jiangsu, China) (37).

The CX-4 automatic biochemical analyzer (Beckman Coulter, Brea, CA, USA) was used to measure the total cholesterol (CHOL), albumin (ALB), glucose (GLU), and blood urea nitrogen (BUN) in serum (38).

### Real-Time Quantitative PCR

The total RNA was extracted from the hypothalamus and pituitary using the TRIzol Reagent (Thermo Fisher Scientific Co., Ltd, CA, USA). Real-time quantitative PCR was performed with a Roche Light Cycler 480II system (Roche Co., Ltd, Basel, Switzerland). Primers (PREMIER Biosoft International, San Francisco, CA, USA) (Supplementary Table 1) were designed using the Primer 5.0 software and synthesized by Sangon Biotech Co., Ltd. (Shanghai, China). The RNA extraction and real-time quantitative PCR were conducted strictly according to previous studies [RT-PCR procedure: step 1: predenaturation program (30 s at 95°C); step 2: PCR (5 s at 95°C for denaturation and 30 s at 60°C for extension); and step 3: dissociation program (5 s at 60°C)] (37, 39, 40). In addition, the relative expression of target genes was calculated by the formula 2^−(ΔΔCt)^ (41).

### Statistical Analysis

In addition to sequencing data, serum indices and mRNA expression were analyzed by the SPSS software (version 20, IBM Corp., Armonk, NY, USA). We filled the missing values with the mean, and the data were logarithmically transformed while outliers existed. Dietary BZN, DON, and their interactions were analyzed using two-way ANOVA. If there was a significant difference, we performed the Bonferroni *t-*test. At the same time, the Duncan's test was used to analyze the significant difference, and the criterion for significance judgment was *p* < 0.05. The GraphPad Prism Software (Version 7; La Jolla, CA, USA) was used to draw the figures, and the column shows mean ± SEM (42–44).

## Results

### Intestinal Microbiome Composition of Piglets

**Figure 1:**
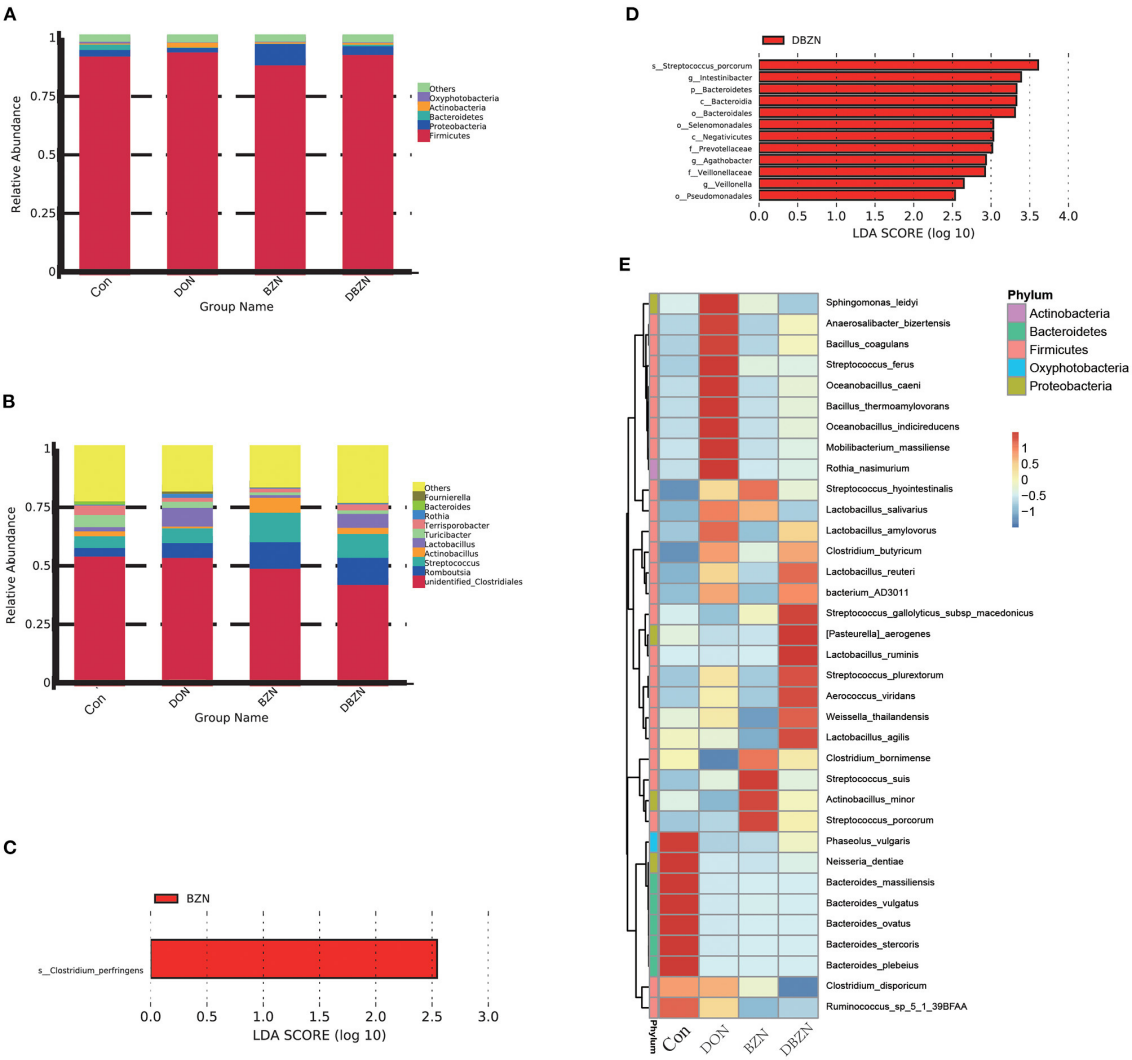
Effects of BZN and DON on intestinal microbiome composition of piglet. **(A)** Intestinal microbiome composition at the phylum level. **(B)** Intestinal microbiome composition at the genus level. **(C)** The LEfSe analysis at different gut microbiota taxa between Con and BZN groups (LDA score >2.5). **(D)** The LEfSe analysis at different gut microbiota taxa between DON and DBZN groups (LDA score >2.5). **(E)** Heatmap showing significantly different species. Dietary treatment: Con, basal diet; DON, 4 mg/kg DON-contaminated diet; BZN, 0.5% BZN supplementation diet; DBZN, 0.5% BZN supplement in 4 mg/kg DON-contaminated diet. BZN, baicalin–zinc complex; Con, control; DON, deoxynivalenol; LDA, linear discriminant analysis.

To characterize the composition of bacterial communities in the ileum, we collected a total of 28 piglets' ileum chymes for 16s sequencing. After quality filtering, 20,694,594 reads that were clustered into 1,096 OTU remained. At the phylum level (Figure 1A), *Firmicutes* was the main phylum in the intestinal microbiota of piglets among the four groups, and the relative *Firmicutes* content in the DON group is much higher (95%). The LEfSe analysis revealed that the relative *Bacteroidetes* content in the DON group was noticeably lower than that in the DBZN group (LDA > 2.5) (Figure 1D). At the genus level (Figure 1B), over 43% of reads were identified as unidentified members of *Clostridiales*. The LEfSe analysis revealed that the relative abundance of *Intestinibacter, Agathobacter*, and *Veillonella* in the DBZN group was noticeably higher than that in the DON group (LDA > 2.5) (Figure 1D). At the species level, for the Con group, intestinal microbiota was mainly enriched in genera belonging to *Bacteroides* (Figure 1C). The BZN group was mainly enriched by *Streptococcus, Clostridium bornimense*, and *Actinobacillus minor*, and the DBZN group was mainly enriched by *Lactobacillus, Streptococcus*, and so on (Figure 1E). The LEfSe analysis showed that BZN markedly increased the relative abundance of *Clostridium perfringens* in basal diet, and BZN markedly increased the relative abundance of *Streptococcus porcorum* in DON-contaminated diet (LDA > 2.5) (Figure 1D).

### Diversity Change of Intestinal Microbiome of the Piglet

**Figure 2:**
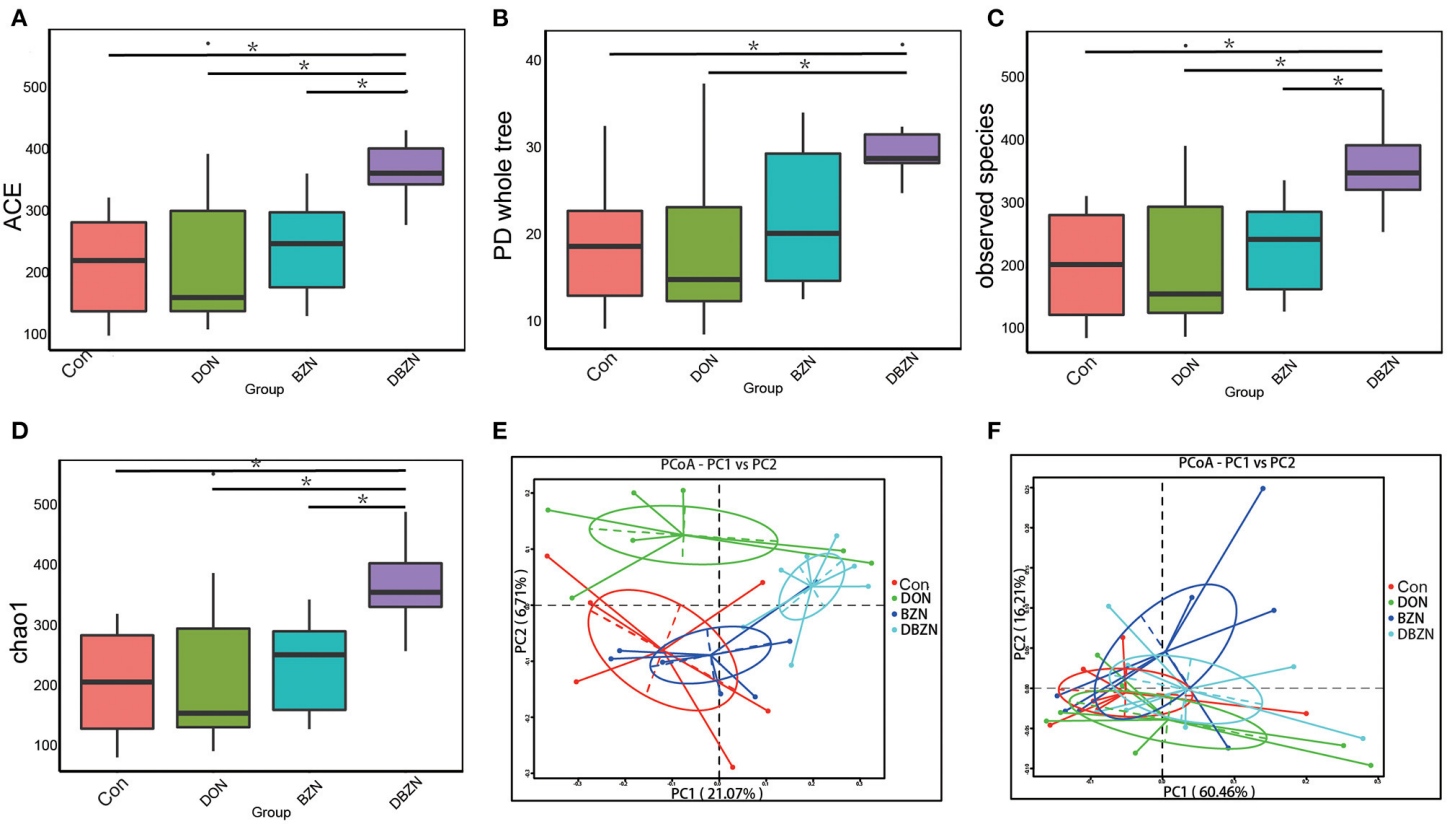
Alpha and beta diversity change of gut microbiota. **(A)** Alpha diversity base on the ACE index. **(B)** Alpha diversity base on the Phylogenetic diversity (PD) whole-tree index. **(C)** Alpha diversity base on the observed species index. **(D)** Alpha diversity base on the chao 1 index. **(E)** Principal coordinate analysis (PCoA) based on the binary Jaccard indices. **(F)** PCoA based on the weighted UniFrac indices. Dietary treatment: Con, basal diet; DON, 4 mg/kg DON-contaminated diet; BZN, 0.5% BZN supplementation diet; DBZN, 0.5% BZN supplement in 4 mg/kg DON-contaminated diet. *p < 0.05. BZN, baicalin–zinc complex; Con, control; DON, deoxynivalenol.

To detect the changes of intestinal microbiota composition during the experiment period, we evaluated the alpha diversity of piglet gut microbiota. The alpha diversity was first calculated using ace, chao 1, PD whole tree, and observed species. All measurements revealed that the BZN supplementation increased evenness and richness of the ileum microbiota in DON-contaminated diet (*p* < 0.05) (Figures 2A–D).

To further reveal the differences in intestinal microbiota composition among the four groups, we evaluated the beta diversity of piglet intestinal microbiota using the binary Jaccard indices and weighted UniFrac (Figures 2E,F). PCoA revealed that although there was no obvious segregation of the BZN group and Con group, the Con group, DON group, and DBZN group can be distinguished from each other. Moreover, analysis of molecular variance (AMOVA) indicated that the differences in the binary Jaccard index between the DON group and DBZN group are significant (*p* < 0.05).

### Metabolic Functional Change of Intestinal Microbiome of the Piglet

**Figure 3:**
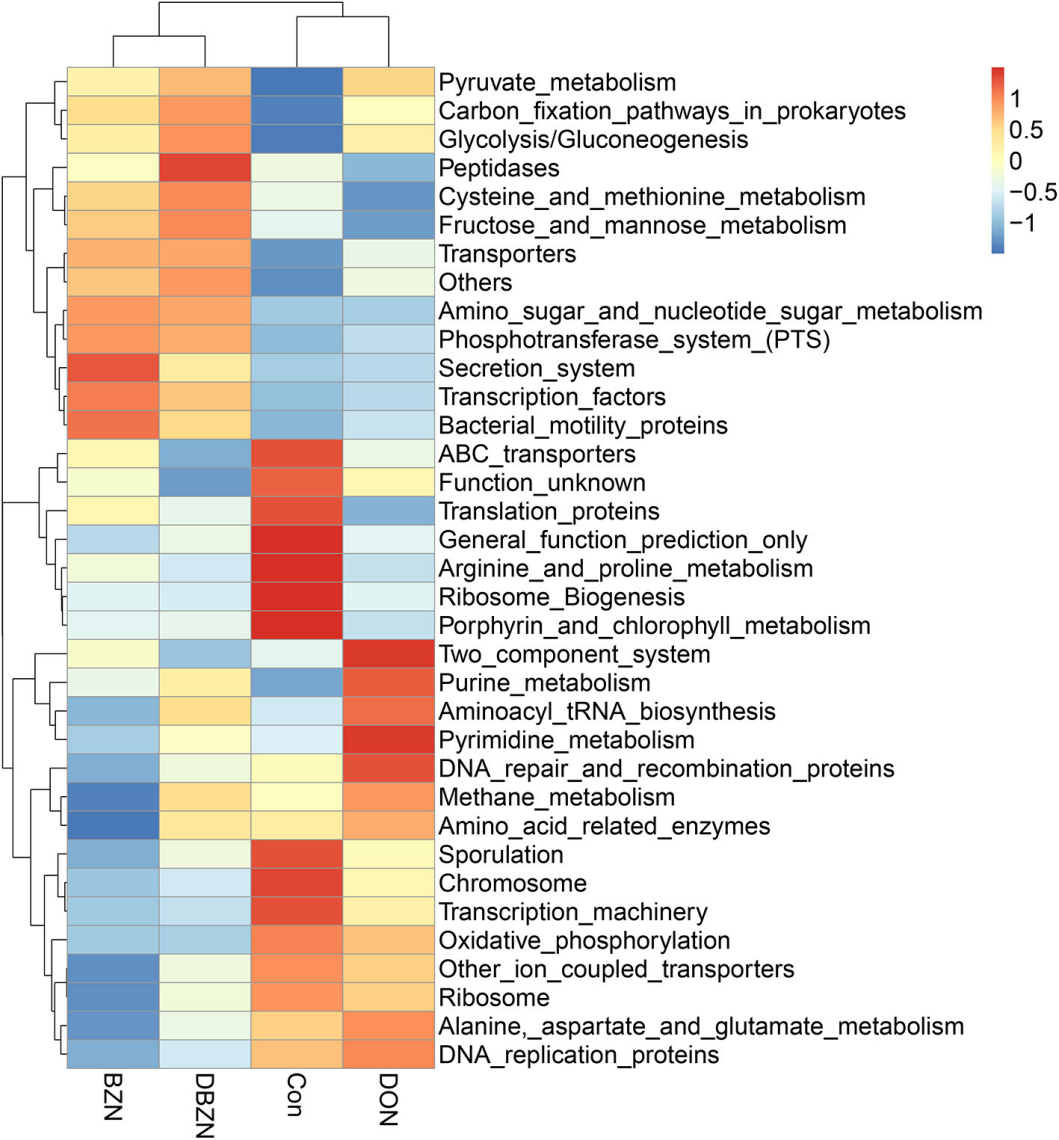
BZN supplementation changed the metabolic functions of intestinal microbiome of piglet at KEGG level 3. Dietary treatment: Con, basal diet; DON, 4 mg/kg DON-contaminated diet; BZN, 0.5% BZN supplementation diet; DBZN, 0.5% BZN supplement in 4 mg/kg DON-contaminated diet. BZN, baicalin–zinc complex; Con, control; DON, deoxynivalenol; KEGG, Kyoto Encyclopedia of Genes and Genomes.

The Phylogenetic Investigation of Communities by Reconstruction of Unobserved States (PICRUST) analysis was used to predict the function of intestinal microbiota (Figure 3). The results indicated that 10 pathways, including ATP-binding cassette (ABC) transporters, translation proteins, general function prediction only, ribosome Biogenesis, porphyrin and chlorophyll metabolism, sporulation, chromosome, transcription machinery, and arginine and proline metabolism were enriched in the Con group. We also identified seven pathways, such as two component system, purine metabolism, aminoacyl tRNA biosynthesis, pyrimidine metabolism, DNA repair and recombination proteins, methane metabolism, and amino acid-related enzymes were predicted to be enriched in the DON group. Seven pathways, including secretion system, transcription factors, bacterial motility proteins, transporters, fructose and mannose metabolism, cysteine and methionine metabolism and peptidases, were predicted to be enriched in the BZN and DBZN groups.

### Serum Inflammatory Responses Correlation With the Intestinal Microbiome Abundance

**Figure 4:**
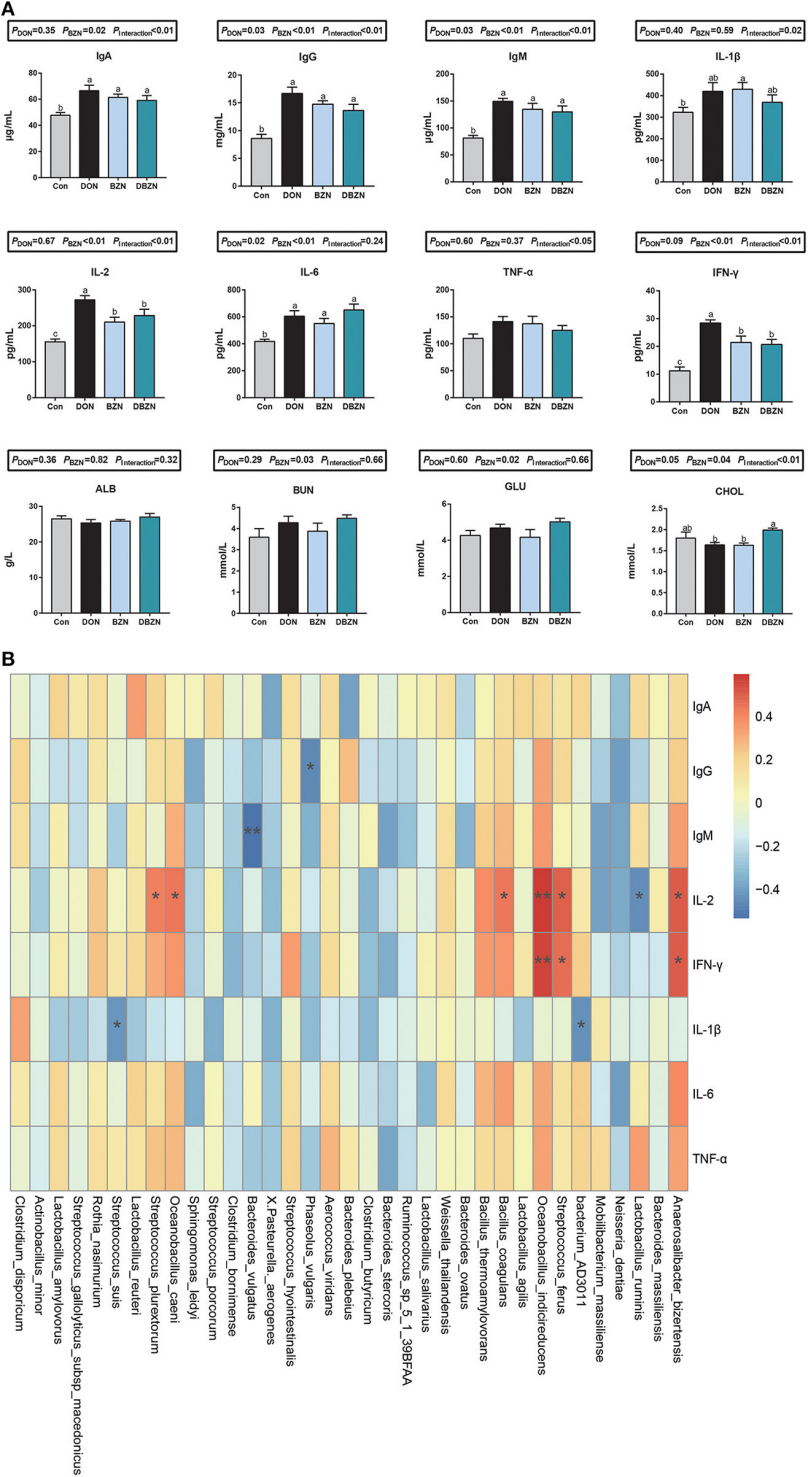
BZN- and DON-mediated inflammatory responses and correlations with intestinal microbiome. **(A)** Serum inflammatory indices. ^ab^Different letters mean significantly difference. **(B)** The Spearman correlations between the intestinal microbiome and serum inflammatory indices. *p < 0.05, **p < 0.01. Dietary treatment: Con, basal diet; DON, 4 mg/kg DON-contaminated diet; BZN, 0.5% BZN supplementation diet; DBZN, 0.5% BZN supplement in 4 mg/kg DON-contaminated diet. IL-1β, interleukin-1β; IgG, immunoglobulin G; IFN-γ, interferon-gamma γ; IL-6, interleukin-6; IgA, immunoglobulin A; GLU, glucose; IgM, immunoglobulin M; CHOL, total cholesterol; IL-2, interleukin-2; TNF-α, tumor necrosis factor-α; ALB, albumin; BUN, blood urea nitrogen; BZN, baicalin–zinc complex; Con, control; DON, deoxynivalenol.

Serum immunoglobulin and cytokines were significantly affected by either supplementation of BZN, DON, or interaction (Figure 4A). Compared with the Con group, the BZN group increased the level of IgA, IgG, IgM, IFN-γ, IL-6, IL-1β, and IL-2 in serum (*p* < 0.05). In the DON-challenged group, the dietary BZN supplementation decreased the level of IL-2 and IFN-γ in serum (*p* < 0.05). The concentration of CHOL in serum was significantly influenced by the interaction effect of DON and BZN (*p* < 0.05). Moreover, the dietary BZN supplementation significantly increased the serum concentration of BUN and GLU in serum (*p* < 0.05).

The Spearman's correlation analysis was used to calculate the relationship between inflammatory and intestinal microbiome abundance. Intestinal microbiota was significantly associated with some immunity indices (Figure 4B). Of these, the abundances of *Bacterium* AD3011, *Phaseolus vulgaris, Bacteroides vulgatus*, or *Streptococcus suis* was negatively correlated to IgG, IgM, and IL-1β, and the abundance of *Anaerosalibacter bizertensis, Streptococcus ferus, Oceanobacillus indicireducens, Bacillus coagulans, Oceanobacillus caeni*, or *Streptococcus plurextorum* was positively correlated to IL-2 and IFN-γ. Moreover, the abundance of *Lactobacillus ruminis* was negatively correlated to IL-2.

### Hormone Secretion Correlation With the Intestinal Microbiome Abundance

**Figure 5:**
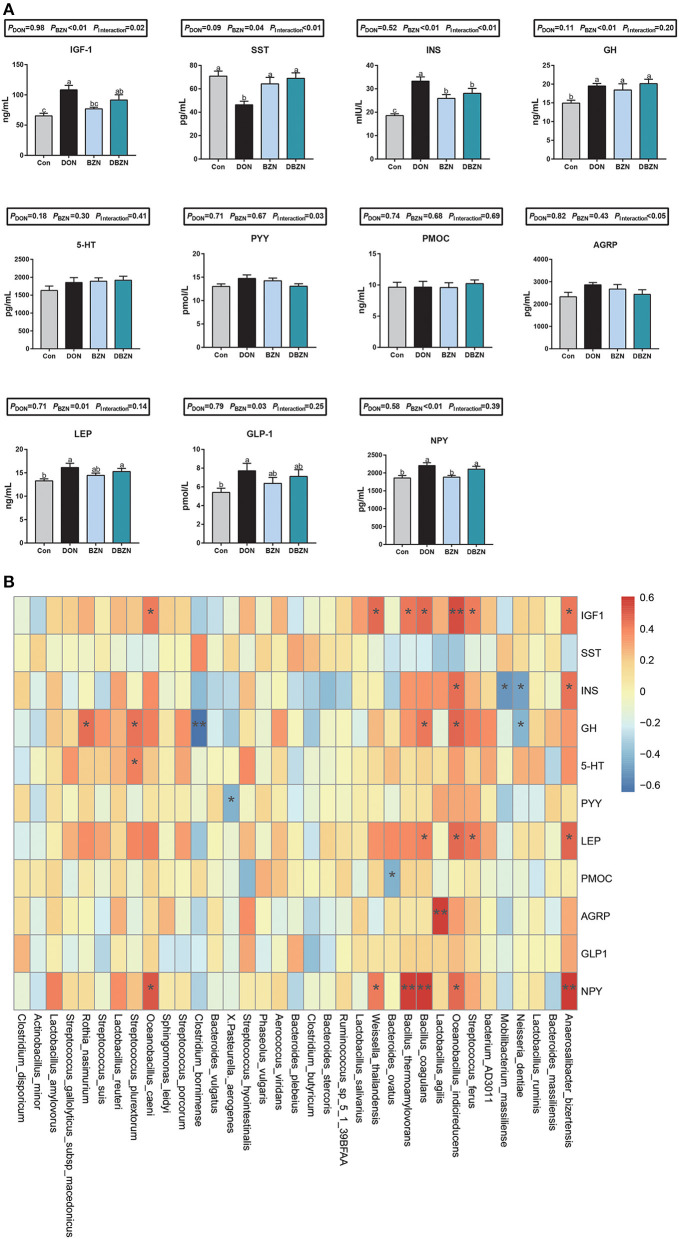
BZN- and DON-mediated hormone secretion and correlations with gut microbiota. **(A)** The concentration of serum hormone. **(B)** The Spearman correlations between the gut microbiota and serum inflammatory makers. ^*^p < 0.05, ^**^p < 0.01. Dietary treatment: Con, basal diet; DON, 4 mg/kg DON-contaminated diet; BZN, 0.5% BZN supplementation diet; DBZN, 0.5% BZN supplement in 4 mg/kg DON-contaminated diet. IGF1, insulin-like growth factor 1; NPY, neuropeptide Y; SST, somatostatin; INS, insulin; GH, growth hormone; 5-HT, 5-hydroxytryptamine; PYY, peptide YY; PMOC, proopiomelanocortin; AGRP, agouti-related protein; LEP, leptin, GLP1, glucagon-like peptide-1; BZN, baicalin–zinc complex; Con, control; DON, deoxynivalenol. ^a,b^different letters means significantly difference (*P* < 0.05).

As shown in Figure 5A, after 2 weeks of experimental treatments, the interaction effect of DON and BZN on GH-related hormones in serum was statistically significant (*p* < 0.01). As compared with the Con group, the concentration of INS and GH of the BZN group was increased (*p* < 0.05). However, the dietary BZN supplementation increased in the concentration of SS, and decreased the concentration of INS in DON-challenged group (*p* < 0.05). As revealed in Figure 5, the concentration of PYY, AGRP, LEP, GLP-1, and NPY in serum was significantly affected by either supplementation of BZN, DON, or interaction (*p* < 0.05).

The Spearman's correlation analysis was used to calculate the relationship between hormone secretion and bacterial genera abundance (Figure 5B). First, the abundance of *Lactobacillus agilis* were positively correlated to the AGRP concentration in serum. As the heatmap shows, the abundance of *Neisseria dentiae, Mobilibacterium massiliense, Bacteroides ovatus, Pasteurella aerogenes*, or *Clostridium bornimense* was negatively correlated to the INS, GH, PYY, and PMOC concentration in serum, and the abundance of *Anaerosalibacter bizertensis, Streptococcus ferus, Oceanobacillus indicireducens, Lactobacillus agilis, Bacillus coagulans, Bacillus thermoamylovorans, Weissella thailandensis, Oceanobacillus caeni, Streptococcus plurextorum*, or *Rothia nasimurium* was positively correlated to the IGF-1, INS, GH, LEP, AGRP, and NPY concentration in serum.

### Relative mRNA Expression in Hypothalamus and Pituitary

**Figure 6:**
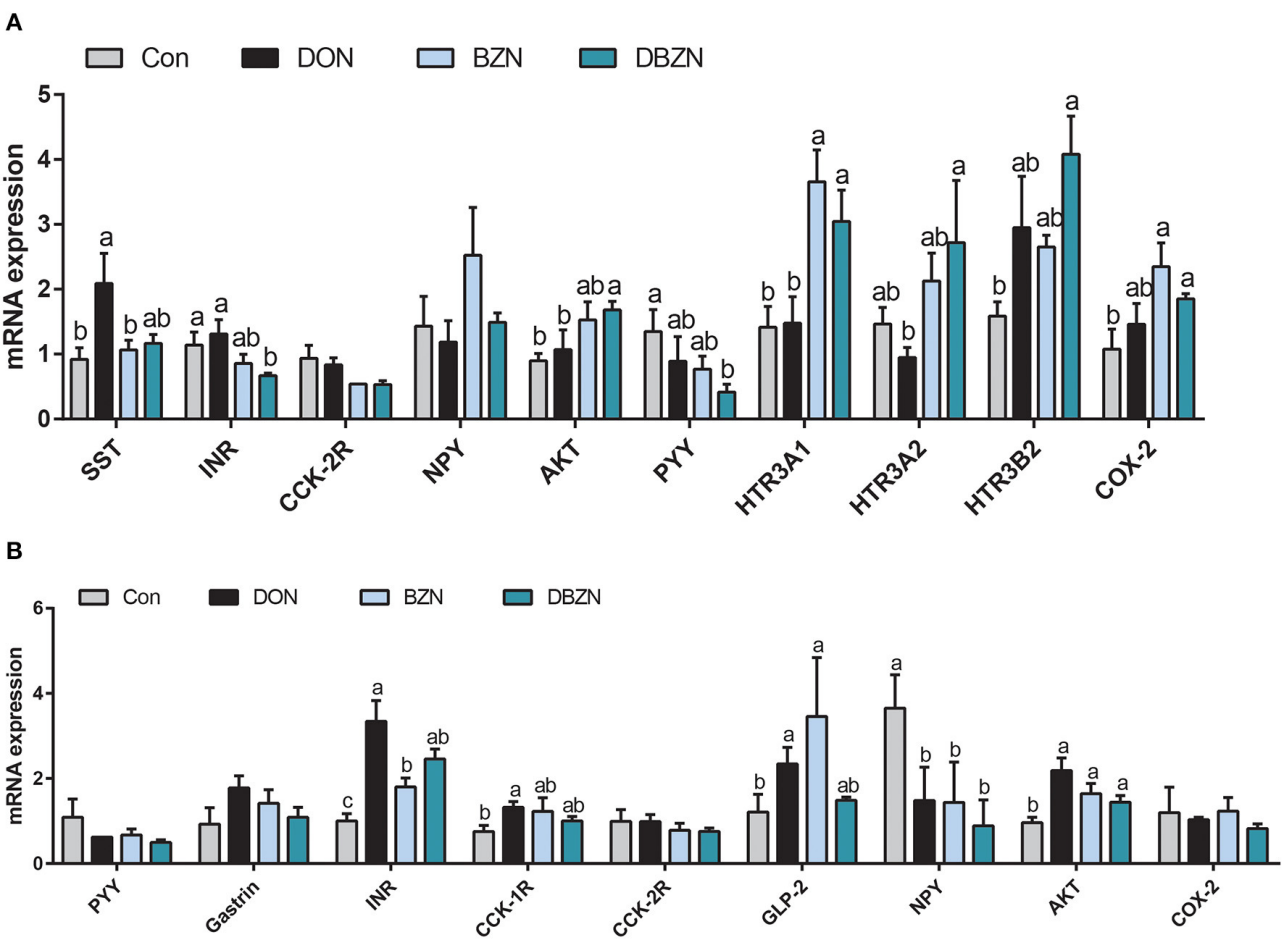
Effect of DON and BZN in mRNA levels of hormone genes in hypothalamus **(A)** and pituitary gland **(B)**. Column was mean ± SEM (*n* = 7). Different letters mean significant differences. Dietary treatment: Con, basal diet; DON, 4 mg/kg DON-contaminated diet; BZN, 0.5% BZN supplementation diet; DBZN, 0.5% BZN supplement in 4 mg/kg DON-contaminated diet. SST, somatostatin; INR, insulin receptor; CCK-2R, cholecystokinin B receptor; NPY, neuropeptide Y; AKT, AKT serine/threonine kinase 1; HTR3A1, 5-hydroxytryptamine receptor 3A 1; HTR3A2, 5-hydroxytryptamine receptor 3A 2; HTR3B2, 5-hydroxytryptamine receptor 3B; COX-2, cyclooxygenase-2; CCK-1R, cholecystokinin type A receptor; GLP-2, glucagon-like peptide 2; BZN, baicalin–zinc complex; Con, control; DON, deoxynivalenol. ^a,b^different letters means significantly difference (*P* < 0.05).

As shown in Figure 6, among the 13 hormones or their receptor genes assayed in the hypothalamus, eight genes were affected by dietary DON, BZN, or their interaction. Specifically, dietary DON exerted a main effect on the mRNA relative levels of insulin receptor (INR), AKT, 5-hydroxytryptamine receptor (HTR)3A1, HTR3A2, HTR3B2, and cholecystokinin (CCK)-2R (*p* < 0.05). The BZN supplementation exerted the main effect on the mRNA levels of SST and HTR3B2 (*p* < 0.05), whereas there were no genes significantly influenced by an interaction between the DON supplementation and the BZN supplementation (*p* > 0.05). Among the 14 hormones or their receptor genes assayed in the pituitary, four genes were affected by dietary DON, BZN, or their interaction. Specifically, dietary DON exerted a main effect of NPY, and the mRNA expression of INR and AKT in the pituitary was affected by dietary BZN (*p* < 0.05). Notably, the mRNA levels of INR, GLP-2, and AKT in the pituitary were influenced by the interaction between the DON supplementation and the BZN supplementation (*p* < 0.05).

## Discussion

Deoxynivalenol is the most common mycotoxin in grains and its products, and it can cause growth retardation, anorexia, and immune abnormalities. In addition, DON also changes the composition of intestinal microbiota, and intestinal microbiota is closely associated with growth and immunity (17, 45). This study showed the impact of BZN supplementation on the intestinal microbiome composition, inflammatory, and hormone secretion in DON-challenged piglets.

Intestinal microbiota is the primary target of DON in animals. Dietary ingestion of DON impaired intestinal homeostasis and changed the composition of intestinal microbiome in mice, especially, DON reduced the abundance of *Streptococcus* (46, 47). Moreover, baicalin regulates intestinal microbiota homeostasis and participates in liver–gut axis interaction (48, 49). Our study showed that dietary BZN in DON-contaminated diet increased intestinal microbial richness and diversity of piglets. Increased intestinal microbial diversity indicates a better resistance to stress. This is similar to the argument that baicalin can reverse intestinal microbiota dysfunction in rats. Moreover, our results showed that there was a significant difference in beta diversity between DON and DBZN groups, which means that dietary BZN in DON-contaminated diets changed the intestinal microbiota structure of piglet. Heatmaps showed that *Lactobacillus ruminis* and *Lactobacillus agilis* were enriched in the DBZN group, and they were able to inhibit *Escherichia coli*, which means BZN may treat intestinal pathogen infection (50, 51). PICRUST showed that the dietary BZN supplementation in DON-contaminated diet enriched peptidases pathway and dietary BZN supplementation in basal diet-enriched secretion system and bacterial mobility protein pathway. In conclusion, the dietary BZN supplementation in DON-contaminated diet relieved the imbalance of the intestinal microbiome caused by DON.

Deoxynivalenol also induced the activation of nuclear factor kappa B (NF-κB) and trigger an inflammatory response, thus selectively inducing the expression of some genes, includes a series of cytokines, chemokines, and other inflammatory factors (17, 52). Studies have also shown that with a high dose of DON, the total serum IgA, IgG, and IgM levels are significantly different from those in the Con group (13, 53). Studies have shown that baicalin treatment can reduce the serum inflammatory biomarkers of spontaneously hypertensive rats, such as IL-6, IL-1β, and high-sensitivity C-reactive protein (hs-CRP). In addition, baicalin treatment can also significantly reduce the expression of toll-like receptor 2 (TLR2), IL-1β, TNF-α, and interleukin-23 (IL-23) in the colon in Spontaneously Hypertensive Rats (SHRs) (54). Baicalin can significantly reduce the serum interleukin-17 (IL-17), IL-6, and IL-1β expression in Ulcerative Colitis (UC) rats (55). In addition, it can also reduce the expression of CD14 and IL-6 in colonic mucosa and alleviate the severity of ulcers (56). The results of these studies have shown that baicalin treatment can inhibit the inflammatory symptoms of the intestinal tract. Our research found that DON and BZN and their interaction groups can make the expression levels of IL-2, IL-6, IgG, IgA, IgM, and IFN-γ significantly different from those of the Con group. Among them, BZN reduced the expression of IL-2 and IFN-γ caused by DON, which was in accordance with the results of a previous study (52). We also observed that the dietary BZN supplementation increased the serum IL-2, IFN-γ, and INS concentration in normal piglets. These results indicate that the effects of DON in normal piglets and DON-induced piglets are different. According to the Spearman correlation analysis, the concentration of IL-2 and IFN-γ was correlated to the abundance of *Anaerosalibacter bizertensis, Streptococcus ferus, Oceanobacillus indicireducens, Bacillus coagulans, Oceanobacillus caeni, Lactobacillus ruminis, or Streptococcus plurextorum*.

Previous literature studies have shown that the growth retardation and anorexia caused by DON may be related to the regulation of hormone secretion, such as 5-HT, GH, and IGF1 (19, 57, 58). Our experiment showed that the dietary BZN supplementation in DON-contaminated diets significantly increased the SS concentration in serum and significantly decreased the INS concentration in serum. SST is a kind of tetrapeptide that can inhibit the secretion of GH and control the secretion of pituitary hormone. It is assumed that the increase of somatostatin concentration will cause growth inhibition. However, it was also noted that the addition of DON and BZN could increase the secretion of GH. Therefore, we conclude that DON unbalanced the growth axis hormone secretion, and the supplementation of BZN can rebalance growth axis hormone secretion induced by DON. Notably, the concentration of GH was positively related to the abundance of *Bacillus coagulans, Oceanobacillus indicireducens, Streptococcus plurextorum*, and *Rothia nasimurium* and was negatively related to the abundance of *Clostridium bornimense* and *Neisseria dentiae*.

## Conclusion

Taken together, these data suggested that BZN was correlated to the change in intestinal microbiota composition and modulated inflammatory and hormone secretion in piglet after the DON exposure. Moreover, the regulation of BZN on inflammation and hormone secretion was related to the change of the abundance of intestinal microbiota.

## Data Availability Statement

The dataset can be found in NCBI (SRA data: PRJNA705396).

## Ethics Statement

The animal study was reviewed and approved by The experiment was carried out under the supervision of the experimental Animal Ethics Committee of the institute of subtropical agriculture (Changsha, Human Province, China).

## Author Contributions

AZ involved in writing—original draft, supervision, visualization, formal analysis, and project administration. PL contributed to methodology, funding acquisition, and project administration. ZC involved in investigation, conceptualization, and formal analysis. SL and MQ contributed to methodology and software. RT analyzed data curation. BT collected resources. All authors contributed to the article and approved the submitted version.

## Funding

This research was supported by Special Funds for Construction of Innovative Provinces in Hunan Province (2019RS3022) and the Joints Funds of the National Science Foundation of China (Grant No: U20A2054).

## Conflict of Interest

The authors declare that the research was conducted in the absence of any commercial or financial relationships that could be construed as a potential conflict of interest.

## Publisher's Note

All claims expressed in this article are solely those of the authors and do not necessarily represent those of their affiliated organizations, or those of the publisher, the editors and the reviewers. Any product that may be evaluated in this article, or claim that may be made by its manufacturer, is not guaranteed or endorsed by the publisher.

## Abbreviations

5-HT: 5-hydroxytryptamine
HTR3A1: 5-hydroxytryptamine receptor 3A 1
HTR3A2: 5-hydroxytryptamine receptor 3A 2
HTR3B2: 5-hydroxytryptamine receptor 3B
AGRP: agouti-related protein
AKT: AKT serine/threonine kinase 1
ALB: albumin
BZN: baicalin–zinc complex
BUN: blood urea nitrogen
CCK: cholecystokinin
CCK-1R: cholecystokinin type A receptor
CCK-2R: cholecystokinin B receptor
CHOL: total cholesterol
COX-2: cyclooxygenase-2
DON: deoxynivalenol
GLP-1: glucagon-like peptide 1
GLP-2: glucagon-like peptide 2
GLU: glucose
GH: growth hormone
hs-CRP: high-sensitivity C-reactive protein
IgA: immunoglobulin A
IgE: immunoglobulin E
IgG: immunoglobulin G
IgM: immunoglobulin M
INS: insulin
INR: insulin receptor
IGF-1: insulin-like growth factor-1
IFN-γ: interferon-gamma
IL-1α: interleukin-1α
IL-1β: interleukin-1β
IL-2: interleukin-2
IL-6: interleukin-6
IL-8: interleukin-8
IL-17: interleukin-17
IL-23: interleukin-23
LEP: leptin
LDA: linear discriminant analysis
NPY: neuropeptide Y
NF-κB: nuclear factor kappa B
OTUs: operational taxonomic units
PYY: peptide YY
PCA: principal component analysis
PCoA: principal co-ordinates analysis
PMOC: proopiomelanocortin
SST: somatostatin
TLR-2: toll-like receptor 2
TNF-α: tumor necrosis factor-α.
