# Supplementary material for: Baicalin–Zinc Complex Alleviates Inflammatory Responses and Hormone Profiles by Microbiome in Deoxynivalenol Induced Piglets
Source: Front Nutr. 2021 Oct 8;8:738281. doi: 10.3389/fnut.2021.738281 (PMC8534294; doi:10.3389/fnut.2021.738281)
Supplement: Supplementary file 1 [file Table_1.docx]

Supplementary Material

Supplemental Table 1. Composition and nutrient level of the diets.

| Ingredients | Content (%) | Nutrient levels | Content (%) |
| --- | --- | --- | --- |
| Corn | 63.70 | DE, MJ/kg | 14.60 |
| Soybean meal | 19.80 | CP, % | 20.27 |
| Whey dried | 4.30 | Calcium, % | 0.69 |
| Fish meal | 9.00 | Phosphorus, % | 0.57 |
| Soybean oil | 0.80 | Lysine, % | 1.26 |
| Lysine | 0.38 | Threonine, % | 0.76 |
| Methionine | 0.10 | Met + Cys, % | 0.62 |
| Threonine | 0.09 | Tryptophan, % | 0.20 |
| Tryptophan | 0.01 | Arginine, % | 1.09 |
| Limestone | 0.52 | Histidine, % | 0.44 |
| NaCl | 0.30 | Isoleucine, % | 0.71 |
| Premix | 1.00 | Leucine, % | 1.52 |
|  |  | Phenylalanine, % | 0.81 |
|  |  | Valine, % | 0.72 |

*The premix feed contain Cu 5 mg; Se 0.3 mg; I 0.1 mg; Fe 80 mg; Zn 85 mg; Mn 3 mg; Vitamin B_1_ 1 mg; Vitamin B_2_ 3 mg; Vitamin B_3_ 12.5 mg; Vitamin B_6_ 1.6 mg; Vitamin B_5_ 10 mg; Vitamin A 2000 IU; Vitamin B_12_ 0.016 mg; Vitamin D_3_ 200 IU; Vitamin E 12 IU; Vitamin K 0.5 mg; folic acid 0.3 mg; Choline chloride 0.5 mg; Vitamin B_7_ 0.05 mg.

Supplemental Table 2. Primers used for Real-time q-PCR.

| Gene | Primer sequence (5'- 3') | Accession number | Size (bp) | Tm (℃) |
| --- | --- | --- | --- | --- |
| PYY | AGATATGCTAATACACCGAT | [XM_021066092.1](https://www.ncbi.nlm.nih.gov/entrez/viewer.fcgi?db=nucleotide&id=1191818649) | 93 | 60.00 |
|  | CCAAACCCTTCTCAGATG |  |  |  |
| SST | CTCTCCATCGTCCTGGCTCT | [NM_001009583.1](https://www.ncbi.nlm.nih.gov/entrez/viewer.fcgi?db=nucleotide&id=57528037) | 159 | 60.00 |
|  | GTTCTCTGTCTGGTTGGGTTCAG |  |  |  |
| INR | GGCATGGTGTACGAGGGAAA | [XM_021083943.1](https://www.ncbi.nlm.nih.gov/entrez/viewer.fcgi?db=nucleotide&id=1191858730) | 124 | 60.00 |
|  | AGGCCTCGTTGAGAAACTCG |  |  |  |
| CCK-1R | GTGGTCCACAGCCTTCTTAT | [XM_021101084.1](https://www.ncbi.nlm.nih.gov/entrez/viewer.fcgi?db=nucleotide&id=1191903242) | 68 | 60.00 |
|  | TCATTTTCGATCCCCAGTT |  |  |  |
| CCK-2R | GCGGCGATCTTTCTGATGAG | [XM_021062350.1](https://www.ncbi.nlm.nih.gov/entrez/viewer.fcgi?db=nucleotide&id=1191907832) | 97 | 60.00 |
|  | GCAGGAAGGCGTTGGTGA |  |  |  |
| GLP-1R | TACTTCTGGCTGCTGGTGGAG | [NM_001256594.1](https://www.ncbi.nlm.nih.gov/entrez/viewer.fcgi?db=nucleotide&id=375268769) | 105 | 60.00 |
|  | ACCCCAGCCTATGCTCAGGTA |  |  |  |
| GLP-2R | TGTCCTACGTGTCGGAGATGTC | [XM_021066117.1](https://www.ncbi.nlm.nih.gov/entrez/viewer.fcgi?db=nucleotide&id=1191818700) | 76 | 60.00 |
|  | TAATTGGCGCCCACGAA |  |  |  |
| c-Fos | CGTGGAGCCAGTCAAGAAC | [NM_001123113.1](https://www.ncbi.nlm.nih.gov/entrez/viewer.fcgi?db=nucleotide&id=178057281) | 157 | 60.00 |
|  | CTCCCAGTCTGCTGCATAG |  |  |  |
| AGRP | GCAGGCCGAGGCCAA | [XM_021093546.1](https://www.ncbi.nlm.nih.gov/entrez/viewer.fcgi?db=nucleotide&id=1191883862) | 57 | 60.00 |
|  | CGTGCCTTGCGTCCTTC |  |  |  |
| NPY | TCGGCGTTGAGACATTACATCA | [NM_001256367.1](https://www.ncbi.nlm.nih.gov/entrez/viewer.fcgi?db=nucleotide&id=373432748) | 68 | 60.00 |
|  | GTCTCGGGACTAGATCGTTTTCC |  |  |  |
| POMC | TGCTTGGAAGATGCCGAGAT | [NM_213858.1](https://www.ncbi.nlm.nih.gov/entrez/viewer.fcgi?db=nucleotide&id=47523145) | 177 | 60.00 |
|  | GCGGAGAGATCTGGTTTGCA |  |  |  |
| 5-HT | ACAGGAACAAGATGACCCCT | [NM_001001267.1](https://www.ncbi.nlm.nih.gov/entrez/viewer.fcgi?db=nucleotide&id=47575844) | 277 | 60.00 |
|  | AGGAGGAACGGGATGTAGAA |  |  |  |
| AKT | TGTGGCAGGATGTGTATGAGA | [XM_021081501.1](https://www.ncbi.nlm.nih.gov/entrez/viewer.fcgi?db=nucleotide&id=1191800852) | 188 | 60.00 |
|  | GTAGGAGAACTGGGGGAAGTG |  |  |  |
| HTR3A 1 | GCCCTTCTGGTGATCAGCTT | [XM_003357301.4](https://www.ncbi.nlm.nih.gov/entrez/viewer.fcgi?db=nucleotide&id=1191909248) | 196 | 60.00 |
|  | AGCAGTCATCGGTCTTGGTG |  |  |  |
| HTR3A 2 | CCCCCAGCCTTGCTTTTAGA | [XM_003357301.4](https://www.ncbi.nlm.nih.gov/entrez/viewer.fcgi?db=nucleotide&id=1191909248) | 122 | 60.00 |
|  | AGGGAAGTGGCCATAGGTGA |  |  |  |
| HTR3B 1 | GTGTGCTCCACGAGTCTTCA | [XM_021062866.1](https://www.ncbi.nlm.nih.gov/entrez/viewer.fcgi?db=nucleotide&id=1191909245) | 98 | 60.00 |
|  | GGGTGACTACAACCAGAGGC |  |  |  |
| HTR3B 2 | AGAGCAGCGCTGGAGATTTT | [XM_021062866.1](https://www.ncbi.nlm.nih.gov/entrez/viewer.fcgi?db=nucleotide&id=1191909245) | 78 | 60.00 |
|  | GGCTCACAACATAGGCCAGT |  |  |  |
| COX-2 | AAGCGAGGACCAGCTTTCACCAAA | [NM_214321.1](https://www.ncbi.nlm.nih.gov/entrez/viewer.fcgi?db=nucleotide&id=47523707) | 93 | 60.00 |
|  | GCGCAGTTTATGCTGTCTCTCCAA |  |  |  |
| GAPDH | ACACTCACTCTTCTACCTTTG | [XM_021091114.1](https://www.ncbi.nlm.nih.gov/entrez/viewer.fcgi?db=nucleotide&id=1191877756) | 90 | 60.00 |
|  | CAAATTCATTGTCGTACCAG |  |  |  |
